# Binge drinking and condom negotiation behaviours among adolescent girls and young women living in Cape Town, South Africa: sexual control and perceived personal power

**DOI:** 10.1186/s12889-023-17188-0

**Published:** 2023-11-18

**Authors:** Suzanne E. Singer, Wendee M. Wechsberg, Tracy Kline, Felicia A. Browne, Brittni N. Howard, Tara Carney, Bronwyn Myers, Courtney Peasant Bonner, Dawn Chin-Quee

**Affiliations:** 1 RTI International, Research Triangle Park, Chapel Hill, NC USA; 2 Gillings School of Global Public Health, University of North Carolina at Chapel Hill, Chapel Hill, NC USA; 3 Department of Psychiatry and Behavioral Sciences, Duke University School of Medicine, Durham, NC 27708 USA; 4 Department of Psychology, North Carolina State University, Raleigh, NC USA; 5 Alcohol, Tobacco and Other Drug Research Unit, South African Medical Research Council, Cape Town, South Africa; 6 Department of Psychiatry and Mental Health, University of Cape Town, Cape Town, South Africa; 7 Curtin enAble Institute, Faculty of Health Sciences, Curtin University, Perth, WA Australia

**Keywords:** Adolescent girls and young women, Alcohol use, HIV risk, Sexual control, Personal agency, Condom negotiation, HIV prevention

## Abstract

**Background:**

Condom use among young people in South Africa has declined in recent years and adolescent girls and young women continue to bear the highest incidence of HIV in the country. Young women who have dropped out of school may be more at risk because of traditional gender norms that create substantial power imbalances and a lack of power to negotiate condom use with their male partners, especially when using alcohol and other drugs.

**Methods:**

This study presents an analysis of baseline data provided by 500 adolescent girls and young women (AGYW) from Cape Town communities between November 2016 and November 2018 who were reached for a cluster-randomised trial conducted to assess the efficacy of an evidence-based, young woman-focused intervention seeking to reduce HIV risk and substance use behaviours. The analysis focuses on associations between binge drinking, condom use, and sexual negotiation, including impaired sex (any substance use at last sex).

**Results:**

AGYW who reported frequent condom negotiation with their partners were 8.92 times (95% CI: [4.36, 18.24]) as likely to use a condom when alcohol or other drugs were not used at last sex and 5.50 times (95% CI: [2.06, 14.72]) as likely when alcohol or other drugs were used at last sex (*p* < 0.05). AGYW who reported frequent binge drinking in the past month (*n* = 177) had significantly reduced odds of condom use at last sex, irrespective of whether the sex was impaired (OR 0.60, 95% CI: [0.49, 0.73]) or not impaired (OR 0.69, 95% CI: [0.60, 0.81]).

**Discussion:**

The findings highlight the need for interventions that reach AGYW in South Africa by specifically aiming to educate AGYW about the effect of binge drinking on negotiating power in their relationships, thus providing them with the knowledge and skills to increase agency regarding condom use.

**Trial registration:**

ClinicalTrials.gov Identifier: NCT02974998 (recruitment completed). 29/11/2016.

## Introduction

For decades, South African youth have benefitted from robust government-supported condom distribution and educational programs that promote understanding of the importance of consistent condom use for preventing HIV, other sexually transmitted infections (STIs), and unintended pregnancy [1–3]. Despite the provision of free and available condoms and other barrier protection methods, data show that condom use among young people in South Africa has declined in recent years in parallel with an increase in HIV incidence, especially among adolescent girls and young women (AGYW), who now bear the highest burden of HIV in the country [4–7]. In a recent study, it was found that although cultural gender norms are changing among youth, most condom use has been controlled by continued gender inequity and male control [8].

Nonetheless condom use within heterosexual partnerships is a dyadic behaviour influenced by multiple sociocultural factors. For example, multiple studies have shown that young people who talk about condom use with their partners are significantly more likely to have used a condom during their last sexual experience [1, 5, 9]. Further, an intervention targeting heterosexual couples in South Africa found that communication significantly increased condom use behaviors, but also impacted male partners’ conceptualization of their role as equal partners and supporters [10].

However, AGYW’s likelihood to feel comfortable communicating about their needs with their male partners, including their desire to use condoms, is inherently dependent on sociocultural norms, many of which perpetuate gendered power imbalances, affect their sense of self-efficacy, and influence other behaviours that could put them at risk for HIV [1, 5, 11, 12]. One such behavior is alcohol and other drug (AOD) use, including binge drinking.

Many AGYW, facing societal pressures, expectations, and gender inequality, such as gender violence, may turn to binge drinking as a coping mechanism [13–16]. Social settings where binge drinking is prevalent can further perpetuate their vulnerability, compelling young women to conform to established norms and expectations, limiting their autonomy and perceived agency [16]. Limited access to resources, opportunities, and education can lead to feelings of control and agency over their circumstances, including their relationships, contributing to alcohol use and subsequent binge drinking [16, 17]. As a result, studies have shown that AGYW are less likely to negotiate condom use and other safer-sex behaviours while drinking alcohol [1–3, 5, 18–20]. Further, young women with low educational attainment or who had dropped out of school are more likely to be living with HIV than their peers who were attending school [21]. Agency, as defined in this study, means that AGYW are able to negotiate condom use.

The relationships between perceived agency, financial support between partners in heterosexual relationships, binge drinking, and adolescents’ and young peoples’ sexual behaviours, including condom negotiation, remain under researched [22–24]. As South Africa has the fifth-highest alcohol consumption rate in the world, with the highest prevalence of binge drinking occurring among people aged 18 to 35, understanding the impact of binge drinking on condom negotiation among this population is imperative to the design of interventions to improve condom use and decrease HIV incidence [25–27]. The current study aims to examine associations between binge drinking and condom use and negotiation in relation to other sociocultural factors among AGYW aged 16 to 19 who did not complete high school in Cape Town, South Africa. Based on patterns observed in previous research, our hypothesis is that AGYW who report frequent binge drinking will be less likely to negotiate condom use with their partners [28, 29].

## Methods

### Design

This study analyses baseline data from the Cape Town Young Women’s Health CoOp Study (YWHC), a cluster-randomised trial that assessed the efficacy of a young-woman–focused intervention relative to treatment as usual for addressing substance use, victimisation, and sexual risk for HIV among AGYW in Cape Town. The specific methods are described in detail elsewhere [30].

### Participants and eligibility

Participants were recruited from 24 target communities (clusters), 12 of which primarily comprised populations self-identifying as Coloured or of mixed racial or ethnic background and 12 of which primarily comprised populations identifying as Black African [31]. These racial categories are representative of Apartheid era classifications that are still used today in South Africa [31].

To be eligible for trial participation, participants (*n* = 500) had to (1) be between 16 and 19 years old at the time of study enrollment, (2) self-identify as female, (3) report consuming 2 to 3 alcoholic drinks at least once in the past month (30 days) *or* report using illicit drugs at least once per week in the past 30 days, (4) be currently dropped out of school, (5) have been out of school for at least 6 months, (6) have not completed high school or a high school equivalent, (7) report condomless sex with a male partner in the past 3 months, (8) reside in one of the 24 target communities for at least 6 months, and plan to live in the target community for at least the next year, and (9) provide verbal and written consent.

### Measures

#### Outcome variables of interest

#### Condom negotiation

Items from the adapted sexual communication scale were used to assess frequency of male and female condom negotiation over the past 6 months. Participants responded to these items on a four-point Likert scale with responses ranging from *(1) Not at all* to *(4) All the time*. Responses of *(3) Many times* or *(4) All the time* to several questions assessing condom negotiation were dichotomously coded as frequent negotiation (1) and all other responses were coded as infrequent negotiation (0). In this analysis, condom use refers to both male and female condom use, as only 3 participants reported asking their partners to use *female* condoms most or all of the time.

#### Condom use during impaired sex

During impaired sex, condom use was assessed using the following items from the same four-point Likert scale: ‘In the past 6 months how often did you use a condom even if you were high or drunk?’ and ‘In the past 6 months how often did you use a condom even if your boyfriend was high or drunk?’ Responses of *(3) Many times* or *(4) All the time* to several questions assessing use of condoms during impaired sex were dichotomously coded as frequent use of condoms during impaired sex (1); all other responses were coded as infrequent use of condoms during impaired sex (0). Responses of *(3) Many times* or *(4) All the time* to these items were coded as frequent use of condoms during impaired sex (1) and responses of *(1)* Not at all or *(2) Response options* were coded as infrequent use of condoms during impaired sex (0).

A single item assessed if participants reported condom use at last sex. Responses for each item were coded as 0 = No and 1 = Yes.

### Covariates

#### Binge drinking

This study defined binge drinking as drinking four or more alcoholic drinks on at least one day in the past month. This aligns with the Substance Abuse and Mental Health Services Administration (SAMHSA) definition of binge-drinking [32].

#### Level of education

Level of education was assessed by asking the question, ‘What is the highest standard/grade that you passed at school?’ Because over half of school dropout occurs after Grade 8 (first year of high school), responses were dichotomised into completed Grade 8 or less (0) and completed Grade 9 to 11 (1).

#### Partners’ financial support

Financial support from partners was assessed through a single item, ‘When you need money and other things, how helpful is your boyfriend?’ Responses of *(2) Somewhat helpful* or *(3) A great deal helpful* were dichotomously coded as financially supported by boyfriend (1); all other responses were coded as not financially supported by boyfriend (0).

#### Power and agency

Power and agency in one’s sexual relationships with their boyfriend was assessed by asking five questions around their perceptions of what would happen if they refused sex with their boyfriend, to which participants responded on a five-point scale ranging from *(1) Strongly agree* to *(5) Strongly disagree*. Scenarios to which participants were asked to respond included a) Sometimes you have sex with your boyfriend even when you do not want to because it is expected of you; b) Sometimes you have sex with your boyfriend even when you do not want to because you are afraid to say NO; c) If you refuse to have sex with your boyfriend, he will refuse to give you money or pay the bills; d) If you refuse to have sex with your boyfriend he will stop giving you his love; and e) If you refuse to have sex with your boyfriend he will become angry or shout at you. Participants’ responses for each item were averaged. Higher average scores reflected more agency and power with their boyfriend. These total scores were recoded into a dichotomous variable: scores ≤ 3 were coded as Low Agency and Power and scores ≥ 3 were coded as High Agency and Power.

#### Length of relationship

A single item assessed how long participants had been dating their boyfriend or person with whom they have regular sex. Participants were asked to provide the duration in days, months, and/or years. Participants were coded as 0 = 6 months or less, 1 = 6 months to 1 year, 2 = 1–2 years, and 3 = More than 2 years.

#### Impaired last sex

A single item assessed whether last sexual encounter was impaired by AOD use. For each question, participants were coded as 0 = No and 1 = Yes. Condom use at last sex was used as a proxy for condom negotiation in this analysis.

#### Analysis

Stata/SE version 16.1 was used for all analyses. Descriptive analyses were performed and the associations among the outcomes of interest (frequency of condom negotiation, frequency of condom use during impaired sex, and condom use at last penetrative sex) and conceptually meaningful covariates (e.g., sociodemographic variables, levels of power and empowerment, length of partnership, financial support from boyfriend or primary partner, and binge drinking) were explored first. Next, separate logistic regression models were conducted to explore the association between any binge drinking in the past 30 days and other variables mentioned above and the frequency of condom negotiation and frequency of condom use during impaired sex. Lastly, separate logistic regression models were conducted to examine the association between the covariates and condom use at last penetrative sex among participants who did and did not report impairment at their last penetrative sex. All analyses accounted for clustering at the neighborhood level using a robust cluster estimator, as neighborhoods are very homogenous with respect to population groups. Thus, we consider that the confounds of population group inequity have been accounted for in the analysis.

## Results

### Sample characteristics

The sociodemographic characteristics of the participants are shown in Table 1. Notably, most participants (77.11%) reported having a boyfriend or someone with whom they regularly have sex, and almost half of participants (47.36%) had been with their boyfriend for 2 years or more. Binge drinking behaviours varied among participants; 177 participants (35.4%) reported drinking 4 or more alcoholic beverages on 4 or more days of the past month and were thus categorised as “binge drinkers”. The mean number of binge drinking days reported by all participants was 5.76 (SD: 7.67). Participants reported a median of 3 days of binge drinking in the past 30 days; however, binge drinking days ranged from 0 to 30 days (in the past month), and the interquartile range (IQR) was 7. Interestingly, 339 participants (86.92%) reported high levels of power and empowerment, and a similar proportion (89.24%) reported receiving financial support from their boyfriend when they needed help. Further, most (79.0%) participants reported having had condomless sex at their last sexual encounter, and some participants (31.0%) reported AOD use just before or during their last sexual encounter. The mean age of participants was 17.76 years old, and most participants (69.80%) had completed schooling up to grades 9 to 11.

**Table 1:** AGYW from Cape Town communities between November 2016—2018 enrolled in a cluster-randomised trial to reduce HIV risk and substance use behaviours at baseline (*n* = 500)

Baseline characteristic	n (%)
Age, mean (SD; range 16–19)	17.76 (1.05)
**Race/population group**	
Black African	246 (49.20)
Coloured	254 (50.80)
**Highest level of education completed**	
Grade 8 or less	151 (30.20)
Grade 9 to 11	349 (69.80)
**Current relationship status**	
Single, never married and not currently involved with a boyfriend	103 (20.68)
Have a boyfriend (or someone to regularly have sex with)	384 (77.11)
Married	6 (1.20)
Separated	5 (1.00)
**How long have you been with your partner? (*n* = 397)**	
Less than 6 months	61 (15.37)
At least 6 months, but less than 1 year	46 (11.59)
At least 1 year, but less than 2 years	74 (18.64)
2 years or more	216 (54.41)
**Financial support received from partner**	365 (89.24)
**High power and empowerment**	339 (86.92)
**Sexual risk**	
Condomless last sex	395 (79.00)
Impaired last sex	155 (31.00)
**Binge drinking behaviours**	3 (7, 30)
Days of binge drinking in the past month (mean, SD)	5.76 (7.67)
Binge drinking in the past 30 days	177 (64.60)

### Variables associated with condom negotiation and use during impaired sex

As shown in Table 2, significantly decreased odds of condom negotiation were found for participants who reported binge drinking in the past 30 days relative to participants who did not report binge drinking in the past 30 days (OR 0.69). Similarly, participants who had been with their boyfriend for 2 years had significantly reduced odds of using condoms during impaired sex (OR 0.70) relative to women in newer partnerships. In contrast, the odds of condom use during impaired sex (OR 1.23, 95% CI: [1.05, 1.45]) increased significantly among participants with high empowerment scores relative to participants with low empowerment scores.

**Table 2:** Impaired sex: baseline odds of condom negotiation and condom use among AGYW from Cape Town communities between November 2016—2018 enrolled in a cluster-randomised trial to reduce HIV risk and substance use behaviours (*n* = 500)

	**Condom negotiation**	
**Value**	**Frequent condom negotiation**	**Frequent use of condoms during impaired sex**
**Binge drinking in the past 30 days**		
OR (95% CI)	0.69 (0.67, 0.72)**	0.75 (.29, 1.97)
**Financial supported by main partner**		
OR (95% CI)	0.61 (0.26, 1.40)	0.56 (0.23, 1.36)
**Partnered for 2 years or more**		
OR (95% CI)	0.68 (0.43, 1.09)	0.70 (0.59, 0.90)*
**High levels of power and empowerment**		
OR (95% CI)	1.03 (0.80, 1.34)	1.23 (1.05, 1.45)*

^**^p < 0.001

^*^p < 0.05

The odds of using a condom at last sex for participants who reported frequent condom negotiation with their partners in the past 6 months were 8.92 times (95% CI: [4.36, 18.24]) the odds of participants who did not report frequent condom negotiation when AODs were not used at last sex, and 5.50 times (95% CI: [2.06, 14.72]) the odds when AODs were used at last sex (p < 0.05) (see Table 3). Participants who reported frequent binge drinking in the past month (*n* = 177) had significantly reduced odds of condom use at last sex irrespective of whether the sex was impaired (OR 0.60, 95% CI: [0.49, 0.73]) or not impaired (OR 0.69, 95% CI: [0.60, 0.81]).

**Table 3:** Last penetrative sex: baseline odds of condom use among AGYW from Cape Town communities between November 2016—2018 (*n* = 500)

	**Condom use during last penetrative sex**	
**Value**	**Not impaired last sex** **(*n* = 345)**	**Impaired last sex** **(*n* = 155)**
**Frequent condom negotiation in the past 6 months (*n* = 55)**		
OR (95% CI)	8.92 (4.36, 18.24)**	5.50 (2.06, 14.72)**
**High empowerment scores (*n* = 339)**		
OR (95% CI)	1.95 (0.74, 5.11)	1.26 (0.79, 1.98)
**Partnered for 2 years or more (*n* = 216)**		
OR (95% CI)	0.66 (0.30, 1.48)	0.79 (0.47, 1.56)
**Binge drinking in the past 30 days (*n* = 177)**		
OR (95% CI)	0.69 (0.60, 0.81)**	0.60 (0.49, 0.73)**

^**^p < 0.001

^*^p < 0.05

## Discussion

The study findings suggest that binge drinking behaviours among AGYW are associated with a lower likelihood of condom negotiation with their boyfriends. Consistent with the previous literature, AGYW in more established relationships, including relationships where they are financially dependent on their partner, were less likely than AGYW in newer relationships to consistently negotiate condom use and to use condoms when AOD use is involved [33, 34]. Importantly, AGYW who reported binge drinking at baseline were unlikely to negotiate condom use; consequently, they were also unlikely to have used a condom at last sex, regardless of alcohol use at last sex. The small number of AGYW who consistently negotiated condom use with their boyfriends were much more likely to have used a condom during their last sexual interaction. However, if they used AODs during or immediately before sex, the likelihood of condom use decreased by over 60%. AOD use during or immediately before AGYW’s last sexual interaction was consistently associated with condomless sex for all AGYW in the study, which confirms the study’s hypothesis.

### Findings in context

In South Africa, the co-occurring public health issues of alcohol use disorders and HIV have a significant population-level impact. Specifically, high rates of HIV prevalence are seen among AGYW who may lack the power to negotiate condom use within their relationships [18, 35]. Although the current body of research has examined the relationships between co-occurring alcohol use and condom use, sociocultural factors such as the financial support a young woman receives from her partner, length of the relationship, and feelings of power and agency have not been thoroughly explored when discussing AGYW’s likelihood to negotiate condom use [4, 7, 36]. By fostering personal power and agency and challenging gender norms within their relationships, AGYW can be empowered to make informed choices and to begin to lead more independent lives. This study builds on the previous literature and fills this gap by examining multiple factors hypothesised to impact the likelihood to negotiate condom use and to use condoms while binge drinking [22].

### Limitations

The data for this study came from questions assessed at baseline prior to AGYW’s participation in the Cape Town Young Women’s CoOp, a larger trial examining the efficacy of a young-woman − focused HIV prevention intervention. In the baseline instrument, the assessment period of condom negotiation behaviours was the past 6 months, whereas binge drinking behaviours were measured in the past 30 days. Because this is an analysis of baseline data, it did not take into account life changes or other factors that may contribute to increased binge drinking in the past month, which could have indicated a longer pattern of binge drinking contributing to an unequal power dynamic among AGYW and their boyfriends. Because of the difference in assessment periods of each outcome, direct causality cannot be inferred. Additionally, it is likely that participants who reported binge drinking were also using other drugs or substances that may impact the likelihood of them negotiating condom use.

Further, this analysis was limited only to participants who reported having a boyfriend or someone with whom they regularly have sex. Because of the exclusion of AGYW who report casual sex with multiple partners, this analysis does not investigate the nuances of power, empowerment, and ability to negotiate condom use within AGYW’s casual sexual encounters, which may be more frequent and make them at even great risk of HIV. Consequently, it may be important for future research to investigate the impact of binge drinking behaviours on AGYW’s ability to negotiate condom use during casual sex. In addition, most participants reported receiving financial support from their boyfriends when they needed it, and a similar proportion reported high levels of agency and personal power. However, despite our understanding that equitable, supportive relationships may increase young women’s power and agency, AGYW who received financial support still had reduced odds of both frequent condom negotiation and frequent use of condoms during impaired sex. Further research should focus on the nuances of this financial support in a greater sociocultural context. Additionally, this study created a combined variable estimating condom negotiation behaviours for use of both male and female condoms, as very few participants reported female condom negotiation and subsequent use. Previous studies have found that AGYW may feel more empowered to negotiate female condom use as compared with male condom use, which, coupled with men’s comparative willingness to use a female condom instead of a male condom, could be imperative for improving condom use behaviours [2]. However, use of the female condom has not been as acceptable [2, 37, 38]. Future research could focus on the events of binge drinking on female negotiation because that seems to be the primary concern related to risk behaviours.

### The Future of woman-focused interventions for AGYW

The Women’s Health CoOp and subsequent adaptations of the intervention have been implemented over the course of almost 20 years in South Africa, with each adaptation seeking to provide the education and skills necessary for women to effectively reduce their alcohol use and improve their overall physical and mental health [39]. These adaptations evolved with the changing landscape of South Africa’s ongoing HIV epidemic and advancements in HIV prevention and treatment. Recent adaptations have expanded to include antiretroviral therapy adherence for women living with HIV [40], stigma-reduction training for clinic providers, and most recently PrEP and contraception uptake for effective HIV and pregnancy prevention among AGYW [41].

In recent years, key global health collaborators have joined forces to take significant steps to address the unique HIV risks faced by AGYW in South Africa. In 2014, the United States President’s Emergency Plan for AIDS Relief (PEPFAR) launched the Determined, Resilient, Empow-ered, AIDS-free, Mentored and Safe (DREAMS) Public/Private Partnership. One of the primary goals of the DREAMS partnership is to empower AGYW by promoting safer sex behaviours, including condom provision and promotion and, notably, by promoting PrEP for young women through a layered intervention approach [41, 42]. Because PrEP does not rely on partner involvement to provide protection against HIV acquisition, the distribution of PrEP is an exceptionally feasible strategy of HIV protection among AGYW who may lack the agency in their relationships to negotiate condom use because of multiple social factors, as analysed in this study [41]. Although South African AGYW benefit from the DREAMS program in addition to one of the largest PrEP promotion and distribution programs in the African continent, adherence among this group remains a concern because of many of the same social factors that impact condom negotiation [43].

Given this consideration, an adapted version of this YWHC implemented in Pretoria, South Africa, has added educational elements of PrEP and sexual and reproductive health for both AGYW and clinic staff who play a critical role in ensuring initiation and adherence to both family planning and HIV prevention methods [41]. The YWHC and its predecessors paved the way for innovative, holistic, and empowering HIV prevention and care interventions that specifically target AGYW and the challenges they face. It is imperative that specific strategies to protect AGYW continue to emerge and evolve to reflect the rapidly changing social environment in which AGYW live, work, and learn.

## Conclusions

Understanding the relationship between binge drinking and AGYW’s likelihood to negotiate condom use with their partners is critical to understanding the necessity of interventions, such as the Cape Town YWHC, for empowering this population both economically and socially and to promote condom negotiation and use [1, 36, 44].

The findings highlight how patterns of binge drinking can disempower women in relationships, leading to a lower likelihood of negotiating condom use, consequently putting AGYW at higher risk for transmission of HIV and other STIs and unintended pregnancy. Woman-focused interventions, such as the Cape Town YWHC, must strive to incorporate conversations about the impact of binge drinking on AGYW’s potential empowerment within their sexual relationships, including how to engage in conversations about HIV and pregnancy protection through condom use and other methods, with their male partners. Key program decision-makers need to consider the ways in which alcohol use interacts with other potentially disempowering factors, such as AGYW’s financial dependence on their partners and the length of their relationships. Addressing the intersectionality of the identities that empower or disempower AGYW in their relationships is crucial to understanding and mitigating their unique HIV risk.

## Authors’ information

Not applicable.

## Data Availability

The datasets used and analysed from the study are available from the corresponding author on reasonable request. Ongoing analysis are underway for additional manuscripts and the final outcomes.

## Abbreviations

AGYW: Adolescent girls and young women
AOD: Alcohol and other drug(s)
DREAMS: Determined, Resilient, Empow-ered, AIDS-free, Mentored and Safe
PEPFAR: President’s Emergency Plan for AIDS Relief
YWC: Young Women’s CoOp
YWHC: Young Women’s Health CoOp
WHC: Women’s Health CoOp
